# Construction of a Metabolism-Related Long Non-Coding RNAs-Based Risk Score Model of Hepatocellular Carcinoma for Prognosis and Personalized Treatment Prediction

**DOI:** 10.3389/pore.2022.1610066

**Published:** 2022-05-24

**Authors:** Peichen Zhang, Liping Chen, Shengjie Wu, Bailiang Ye, Chao Chen, Lingyan Shi

**Affiliations:** ^1^ Department of Gastrointestinal Surgery, The First Affiliated Hospital of Wenzhou Medical University, Wenzhou, China; ^2^ Department of Pharmacy, Sir Run Run Shaw Hospital, School of Medicine, Zhejiang University, Hangzhou, China; ^3^ Department of Gastroenterology, The First Affiliated Hospital of Wenzhou Medical University, Wenzhou, China

**Keywords:** prognosis, lncRNA, hepatocellular carcinoma, metabolism, therapeutic response

## Abstract

**Background:** Long non-coding RNAs (lncRNAs) play a key regulatory role in tumor metabolism. Although hepatocellular carcinoma (HCC) is a metabolic disease, there have been few systematic reports on the association between lncRNA expression and metabolism in HCC.

**Results:** In this study, we screened 557 metabolism-related lncRNAs in HCC. A risk score model based on 13 metabolism-related lncRNA pairs was constructed to predict the outcome and drug response in HCC. The risk score model presented a better prediction of the outcomes than that with common clinicopathological characteristics, such as tumor stage, grade, and status and aneuploidy score in both training and testing cohorts. In addition, patients in the high-risk group exhibited higher responses to gemcitabine and epothilone, whereas those in the low-risk group were more sensitive to metformin and nilotinib.

**Conclusion:** The metabolism-related lncRNAs-based risk score model and the other findings of this study may be helpful for HCC prognosis and personalized treatment prediction.

## Introduction

Liver cancer remains a global health challenge, and it is the sixth most frequently occurred cancer, with an increasing incidence worldwide [1, 2]. Hepatocellular carcinoma (HCC) is a very common form of primary liver cancer, accounting for approximately 90% of cases [2]. Surgical resection remains the most effective treatment for HCC. However, most patients are diagnosed in the middle and advanced stages, and less than one-third of patients with HCC are suitable for surgery [3]. In addition, the prognosis of patients undergoing surgical resection remains poor owing to the high recurrence rate; thus, determination of the underlying molecular mechanisms and construction of an effective prognostic model are urgently needed [3].

Similar to all malignant tumors, the growth of HCC is characterized by uncontrolled and rapid proliferation. Based on the Warburg effect, the metabolic characteristics of tumor cells are different from those of normal cells to adapt to their rapid growth and proliferation [4, 5]. Consequently, HCC cells can take up more glucose and tend to depend on aerobic glycolysis, glutamine uptake, and decomposition to rapidly produce adenosine triphosphate, thus promoting macromolecule biosynthesis and maintaining appropriate REDOX homeostasis [4, 5]. These changes in tumor metabolism are mainly regulated by cell growth and proliferation signaling pathways, which in turn regulate the metabolic network through various transcriptional and post-translational regulatory mechanisms [6].

Increasing evidence indicates that aberrant long non-coding RNAs (lncRNAs) are closely related to the occurrence and development of HCC. LncRNAs are a group of endogenous RNAs with lengths >200 nucleotides that lack a specific complete open reading frame and protein coding function [7, 8]. To date, at least 74 lncRNAs have been reported to be deregulated in HCC. For example, lncRNA-HLUC, lncRNA-H19, and lncRNA-CUDR are highly expressed in HCC cells compared with those in normal liver cells [9–12]. At the early stage, the expression level of lncRNA-MALAT-1 increased up to six times that in normal cells [13]. In recent years, further analysis has suggested that lncRNAs play important roles in the regulation of tumor cell metabolism [14]. Moreover, lncRNAs can regulate key steps in glucose, protein, lipid, and nucleic acid metabolism in tumor cells to form tumor cells in a hypermetabolic state and provide the necessary energy and material basis for the survival of tumor cells. Therefore, it is of great clinical value to understand the relationship between lncRNA expression and metabolism in HCC cells and to elucidate the relevant molecular mechanisms for the treatment of HCC.

In this study, we screened 557 metabolism-related lncRNAs in Cancer Genome Atlas (TCGA)-Liver Hepatocellular Carcinoma cohort, among which 105 were differentially expressed between the tumor and normal tissues. To facilitate and broaden the clinical applications in different institutions, we constructed lncRNA pairs. A 13-lncRNA pair-based risk score model was built to predict the outcome and drug response in the training and testing cohorts.

## Methods

### Data Sources and Preparation

The transcriptome data and clinical data of patients with HCC were downloaded from TCGA (https://cancergenome.nih.gov/). We divided the gene expression data into mRNA and lncRNA data according to their annotation. A list of 2752 metabolism-related genes encoding all known human metabolic enzymes and transporters is shown in Supplementary Table S1 [15]. We randomly selected 70% of the patients as the training cohort (*n* = 252), wheras 30% of those as the testing cohort (*n* = 108).

### Identification of Metabolism-Related lncRNAs and lncRNA Pairs

We performed Pearson’s correlation analysis of 2752 reported metabolism-related genes and lncRNAs to identify metabolism-related lncRNAs by setting a correlation coefficient of >0.5 at *p* ≤ 0.0001. Differentially expressed metabolism-related lncRNAs between tumor and normal tissues of HCC were screened out by setting the threshold false discovery rate ≤0.05 at |log2FC| ≥ 2. To broaden their application value, we defined lncRNA pairs using the identified metabolism-related lncRNAs. For instance, the value of the lncRNA pair lncRNA-A|lncRNA-B is 1 if the expression of lncRNA-A is greater than that of lncRNA-B and is 0 otherwise.

### Data Analysis

All statistical analyses were performed using R programming. Differentially expressed lncRNAs were identified by the “limma” package [16]. The least absolute shrinkage and selection operator (lasso) risk score model was constructed using identified metabolic lncRNA pairs using the “glmnet” package. The time-dependent receiver operating characteristic (ROC) curve was plotted using the “survivalROC” package. Survival analysis was performed using the Kaplan–Meier method and log-rank test. In addition, chi-square test was used to compare the association between the risk groups and clinicopathological characteristics. The estimation of infiltrating immune cells was downloaded from http://timer.comp-genomics.org. Gene set enrichment analysis was performed using h.all.v7.2.entrez.xls downloaded from http://www.gsea-msigdb.org/gsea/downloads.jsp [17, 18]. Responses to chemotherapeutic drugs were predicted using “pRRophetic” package [19]. IC_50_ was used to evaluate drug susceptibility. Unpaired two-sided t-test was used to compare two experimental groups.

## Results

### Screening of Differentially Expressed Metabolism-Related Long Non-Coding RNAs in HCC

**FIGURE 1:**
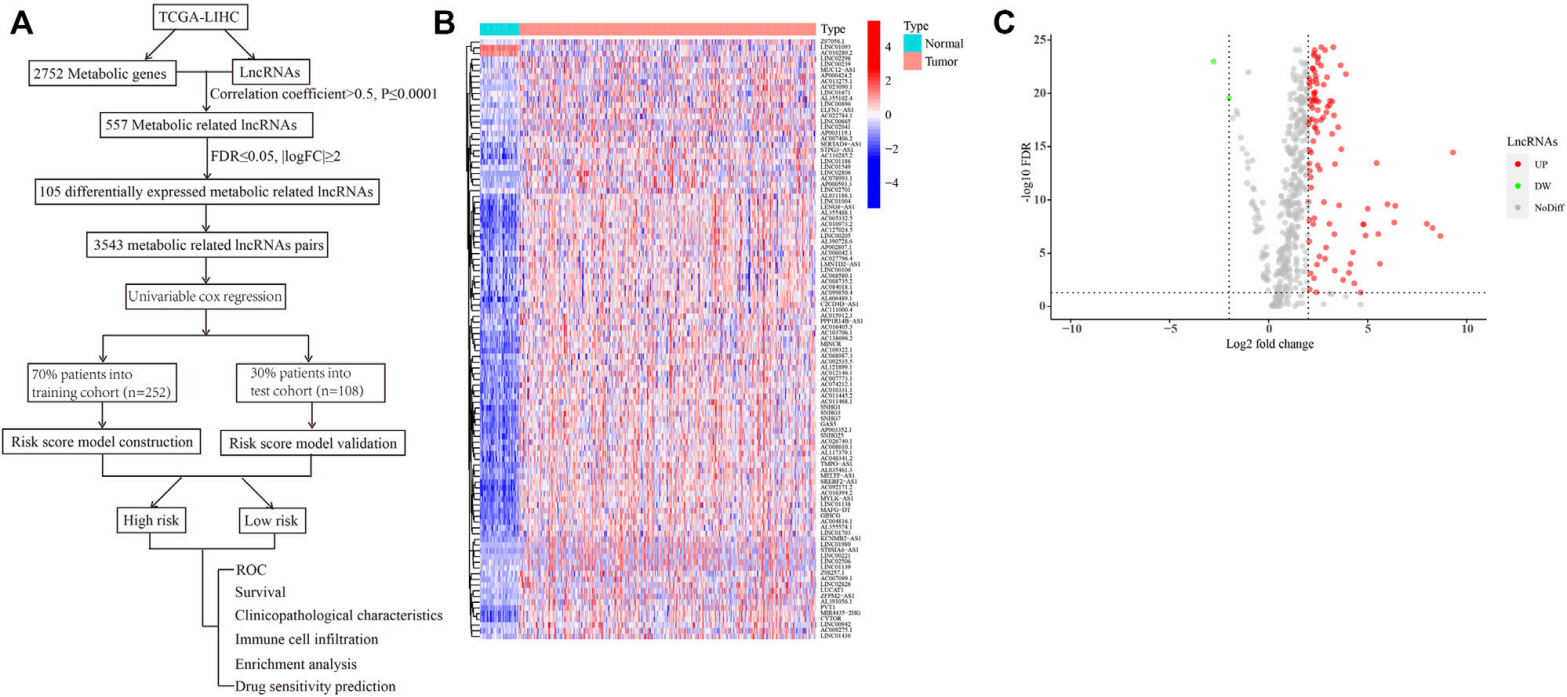
Identification of differentially expressed metabolism-related lncRNAs in HCC. **(A)** A flowchart showing the systematic summary of the study. **(B)** A heatmap displaying differentially expressed metabolism-related lncRNAs between tumor and normal tissues. **(C)** A volcano plot showing the significantly upregulated and downregulated expressions of lncRNAs (false discovery rate ≤ 0.05; |log2FC| ≥ 2).

A flowchart providing a systematic summary of our study is shown in Figure 1A. Using correlation analysis, we identified 557 lncRNAs that may be correlated with metabolism-related genes (correlation coefficient >0.5; *p* ≤ 0.0001; Supplementary Table S2). We then performed differential expression analysis of these metabolism-related lncRNAs between tumor and normal tissues. As shown in Figures 1B,C and Supplementary Table S3, two downregulated lncRNAs and 103 upregulated lncRNAs in HCC were observed (false discovery rate ≤0.05; |log2FC|≥2). To facilitate and broaden the clinical applications in different institutions, we constructed lncRNA pairs (Supplementary Table S4). We identified 3543 lncRNA pairs using the value assignments described above.

### Construction and Testing of a Risk Score Model Using Identified lncRNA Pairs

**FIGURE 2:**
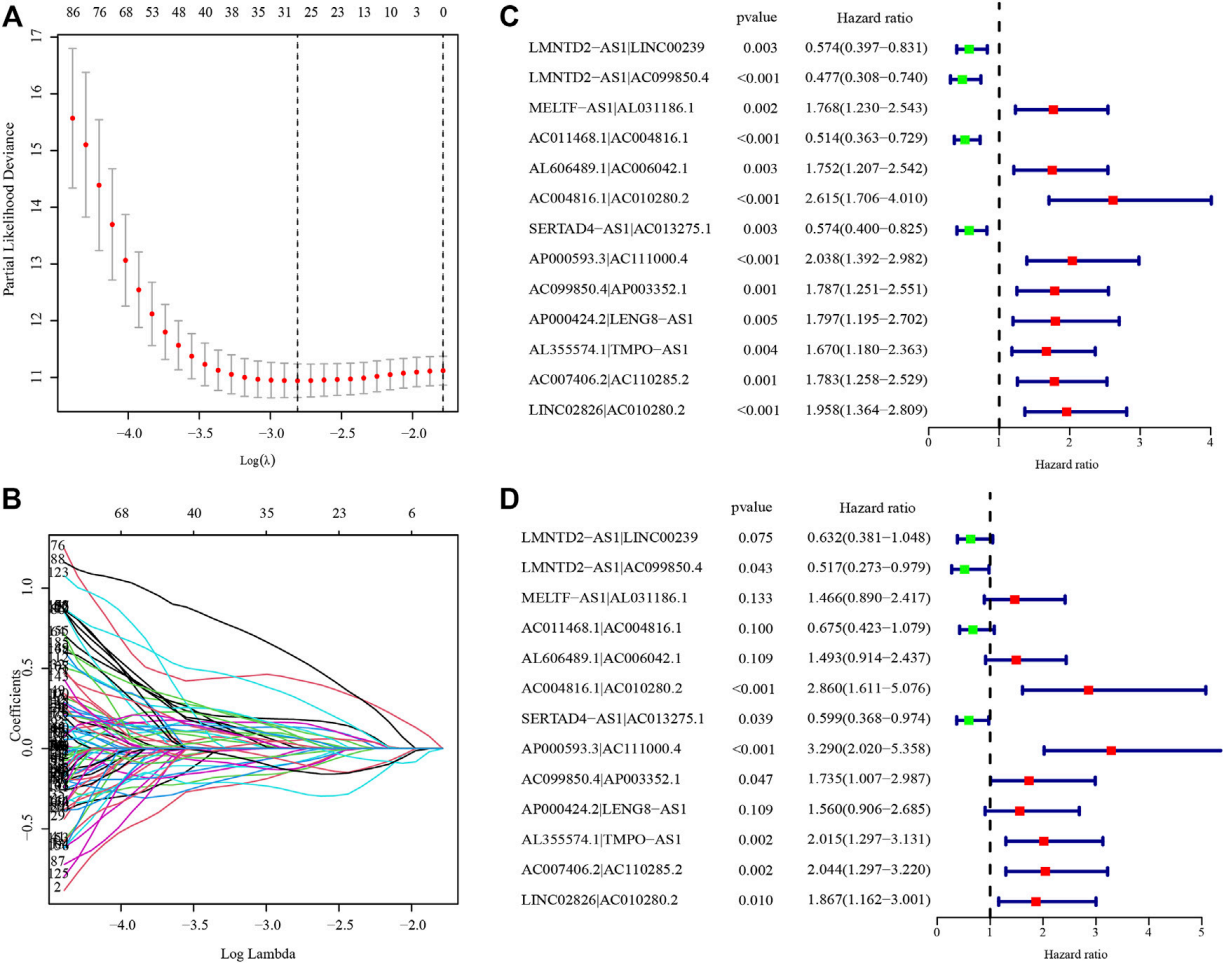
Establishment of the risk score model based on differentially expressed metabolism-related lncRNA pairs. **(A)** The partial likelihood deviances of building the risk score model. **(B)** The solution paths of the risk score model. **(C)** Univariate analysis of lncRNA pairs involved in the risk score model. **(D)** Multivariate analysis of lncRNA pairs involved in the risk score model. The hazard ratio was shown with corresponding 95% confidence intervals (95% CIs).

**FIGURE 3:**
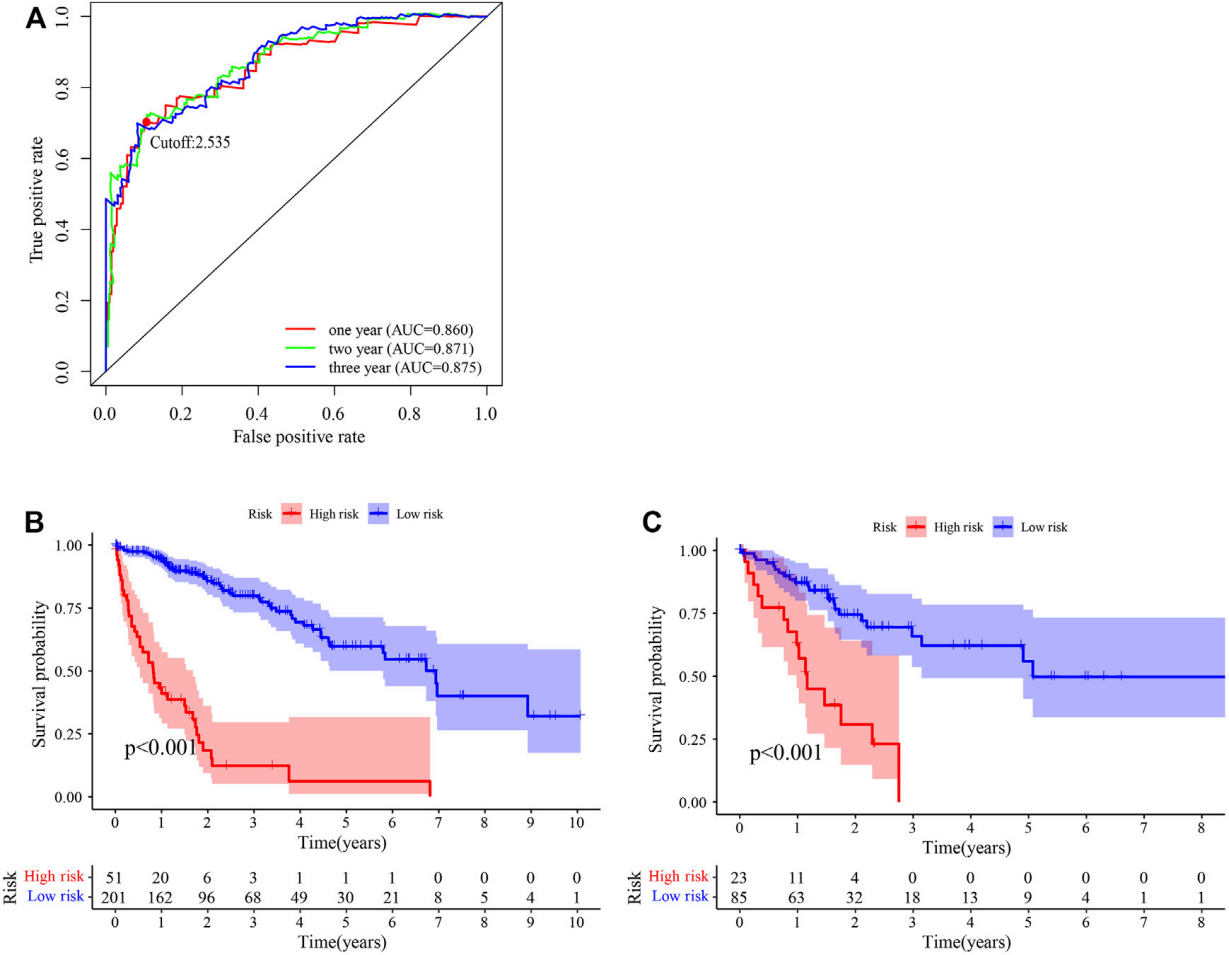
Predictive performance of the risk score model. **(A)** Time-dependent receiver operating characteristic curve of the risk score. **(B, C)** Survival curve of patients with HCC stratified by the risk score model in the training cohort (*n* = 252) **(B)** and testing cohort (*n* = 108) **(C)**.

After identifying 3543 metabolism-related lncRNA pairs, we performed univariate Cox regression analysis of each lncRNA pair. Then, 188 lncRNA pairs with *p* < 0.005 were enrolled as candidate lncRNA pairs in the following construction of a lasso regression model (Supplementary Table S5). A lasso regression model was built using identified lncRNA pairs to predict the relative risk of patients with HCC. As shown in Figure 2, 13 lncRNA pairs were included in the risk score model. The risk score was calculated as −0.46*(LMNTD2-AS1|LINC00239)−0.66*(LMNTD2-AS1|AC099850.4)+0.38*(MELTF-AS1|AL031186.1)-0.39*(AC011468.1|AC004816.1)+0.40*(AL606489.1|AC006042.1)+1.05*(AC004816.1|AC010280.2)-0.51*(SERTAD4-AS1|AC013275.1)+1.19*(AP000593.3|AC111000.4)+0.55*(AC099850.4|AP003352.1)+0.44*(AP000424.2|LENG8-AS1)+0.70*(AL355574.1|TMPO-AS1)+0.71*(AC007406.2|AC110285.2)+0.62*LINC02826|AC010280.2), and the value of an lncRNA pair lncRNA-A|lncRNA-B is described above. The area under the ROC curve (AUC) of 1 year was 0.860, whereas that of 3 years increased to 0.875, which may indicate that the risk score model presented a better ability to predict outcomes over time (Figure 3A). The point closest to the upper left corner was the optimal critical cutoff to identify the high-risk and low-risk groups (cutoff = 2.535, the high-risk group with risk score ≥2.535 or the low-risk group otherwise; Supplementary Table S6). In general, the risk score model had a very strong ability to predict the outcome of HCC in the training and validation cohorts (*p* < 0.001, Figures 3B,C).

### Prognostic Performance of the Risk Score Model in the Training and Testing Cohorts

**FIGURE 4:**
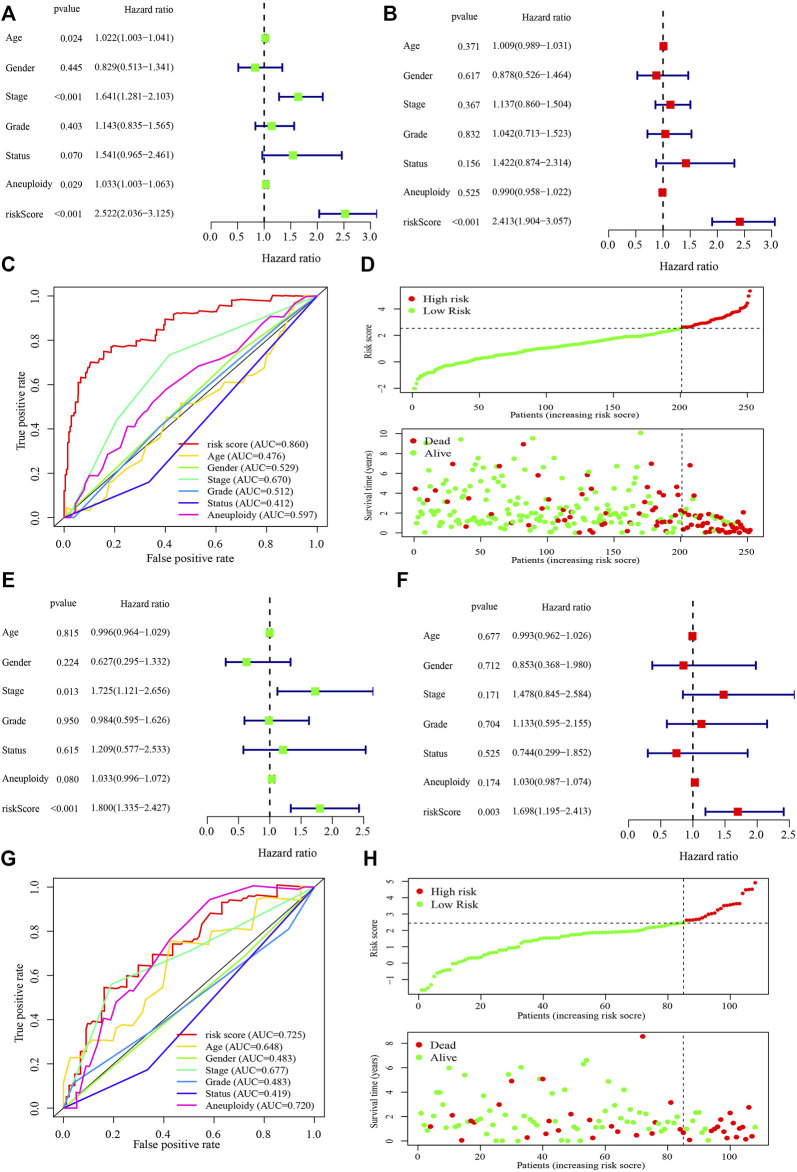
Performance of the risk score model and clinicopathological characteristics in the training and testing cohorts. **(A, B) **Univariate **(A)** and multivariate **(B)** analyses of the risk score model and clinicopathological characteristics in the training cohort. **(C)** Receiver operating characteristic curve of the risk score model and clinicopathological characteristics in the training cohort. (D) The risk score and survival distribution of the high- and low-risk groups in the training cohort. **(E, F)** Univariate **(E)** and multivariate **(F)** analyses of the risk score model and clinicopathological characteristics in the testing cohort. **(G)** Receiver operating characteristic curve of the risk score model and clinicopathological characteristics in the testing cohort. **(H)** The risk score and survival distribution of the high- and low-risk groups in the testing cohort. The hazard ratio was shown with corresponding 95% confidence intervals (95% CIs).

In the independent prognostic analyses of the risk score and clinicopathological characteristics, the risk score was an independent risk factor for both univariate (hazard ratio [HR]: 2.522 (2.036–3.125), *p* < 0.001) and multivariate (HR: 2.413 (1.904–3.057), *p* < 0.001) analyses (Figures 4A,B). Furthermore, the risk score (AUC = 0.860) had a higher prognostic capacity than that of age (AUC = 0.476), gender (AUC = 0.529), tumor stage (AUC = 0.670), tumor grade (AUC = 0.512), cancer status (AUC = 0.412), and aneuploidy score (AUC = 0.597) (Figure 4C). Patients in the high-risk group exhibited higher mortality rates and shorter survival time in the training cohort (Figure 4D). Moreover, in the testing cohort, the risk score was also an independent risk factor for both univariate (hazard ratio [HR]: 1.800 (1.335–2.427), *p* < 0.001) and multivariate (HR: 1.698 (1.195–2.413), *p* = 0.003) analyses (Figures 4E,F). The risk score (AUC = 0.725) had a higher prognostic capacity than that other characteristics such as age (AUC = 0.648), gender (AUC = 0.483), tumor stage (AUC = 0.677), tumor grade (AUC = 0.483), cancer status (AUC = 0.419), and aneuploidy score (AUC = 0.720) in the testing cohort (Figure 4G). And patients in the high-risk group had shorter survival time (Figure 4H).

### Correlation Analyses Between the Risk Groups and Clinicopathological Characteristics in the Training and Testing Cohorts

**FIGURE 5:**
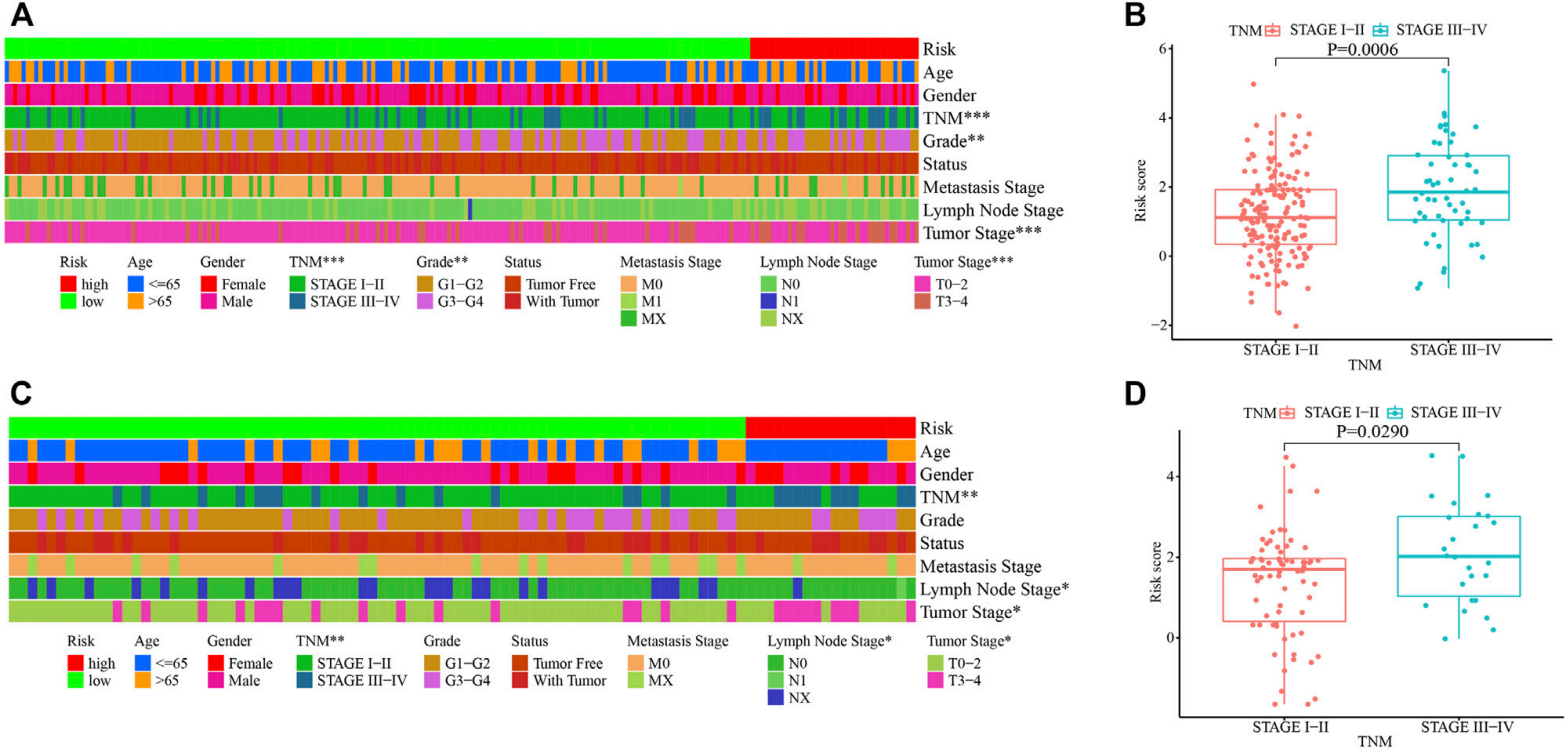
Clinicopathological characteristics of HCC stratified by the risk score model in the training and testing cohorts. **(A, C)** Correlation of clinicopathological characteristics in the high- and low-risk groups in the training **(A)** and testing **(C)** cohorts. **(B, D)** Correlation of TNM staging and the risk score in the training **(B)** and testing **(D)** cohorts. *, *p* < 0.05; **, *p* < 0.01, ***, *p* < 0.001.

Correlation analyses between the risk groups and clinicopathologic features including age, gender, tumor grade, TNM staging, cancer status, metastasis stage, lymph node stage, and tumor stage, were performed. Patients in stages III–IV, grades 3–4, or T3–4 stage had higher risk scores in the training cohort (Figures 5A,B). While in the testing cohort, patients in stages III–IV, or T3–4 stage had higher risk scores in the testing cohort (Figures 5C,D). We then analyzed the infiltrating immune cells in the tumor microenvironment between the low- and high-risk groups using various software (Supplementary Figure S1A and Supplementary Table S7). Among them, CD4^+^ Th2 (*p* < 0.0001), T cell CD4^+^ memory (*p* = 0.0320) and macrophage M0 (*p* = 0.0230) cells were positively correlated in the high-risk group (Supplementary Figures S1B–D).

### Enrichment Analysis and Drug Sensitivity Prediction in the Training and Testing Cohort

**FIGURE 6:**
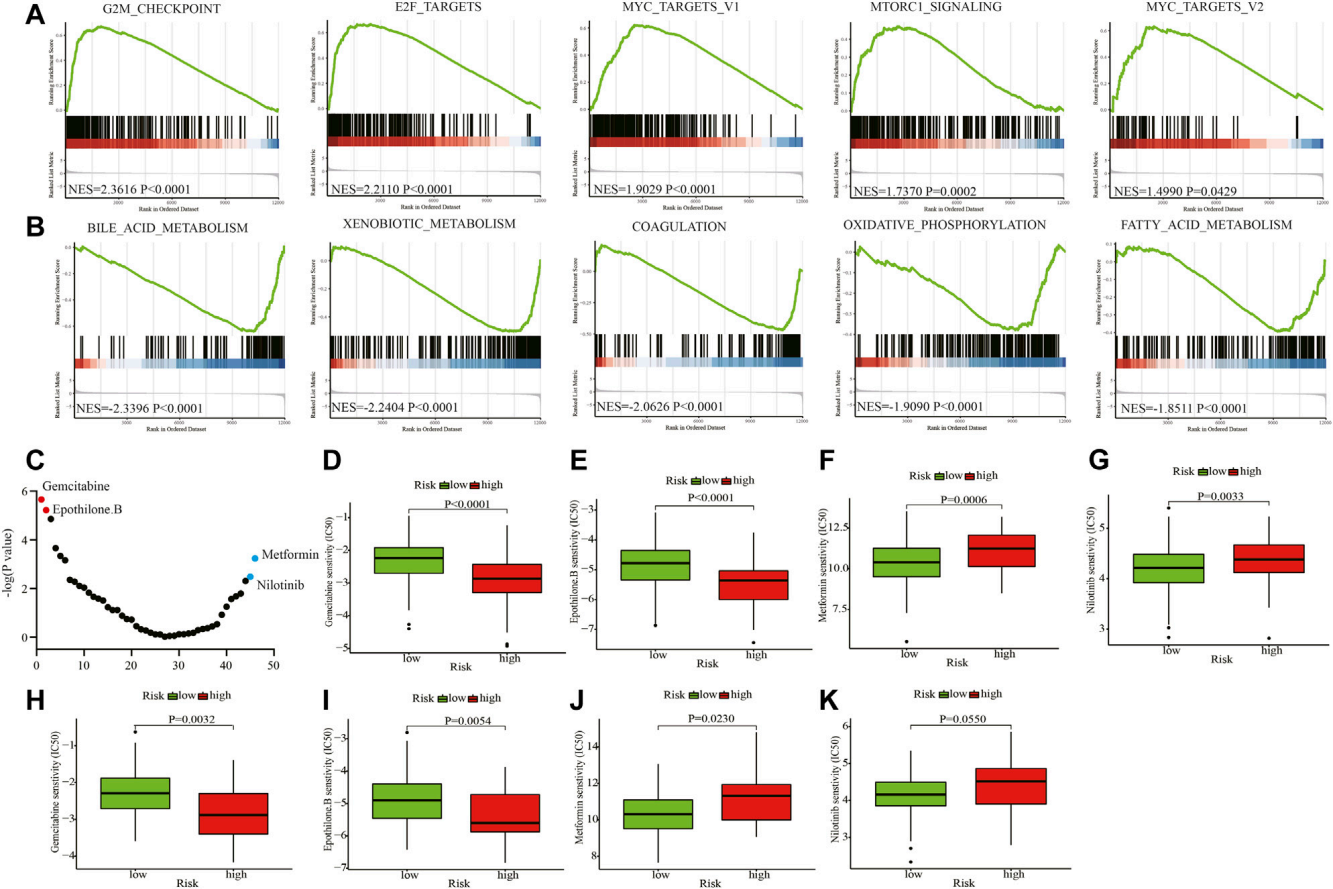
Enrichment analyses and drug sensitivity prediction. **(A, B)** GSEA enrichment plots of the top enriched gene sets of the high-risk **(A)** and low-risk groups **(B)**. **(C)** The differential response to 46 chemotherapy drugs of the two groups. **(D, E)** Patients in the high-risk group may be sensitive to gemcitabine and epothilone B in the training cohort. **(F, G)** Patients in the low-risk group may be susceptible to metformin and nilotinib in the training cohort. **(H, I)** Patients in the high-risk group may be sensitive to gemcitabine and epothilone B in the testing cohort. **(J, K)** Patients in the low-risk group may be vulnerable to metformin and nilotinib in the testing cohort. GSEA: Gene set enrichment analysis; NES: Normalized enrichment score; IC50: half maximal inhibitory concentration.

We conducted enrichment analysis between the high- and low-risk groups using hallmark gene sets in the training cohort. The G2M checkpoint, E2F targets, MYC targets V1, MTORC1, and MYC targets V2-related pathways were significantly enriched in the high-risk group (Figure 6A). Bile acid metabolism, xenobiotic metabolism, coagulation, oxidative phosphorylation, and fatty acid metabolism-related pathways were relatively enriched in the low-risk group (Figure 6B). Using the R package “pRRophetic”, we predicted the clinical chemotherapeutic response to several chemotherapy drugs based on the tumor gene expression between the two groups (Figure 6C and Supplementary Table S8). In the training cohort, patients in the high-risk group exhibited higher responses to the chemotherapeutics gemcitabine (*p* < 0.0001) and epothilone B (*p* < 0.0001) (Figures 6D,E), whereas those in the low-risk group may be more sensitive to metformin (*p* = 0.0006) and nilotinib (*p* = 0.0033) (Figures 6F,G). In the testing cohort, patients in the high-risk group were also more vulnerable to gemcitabine (*p* = 0.0032) and epothilone B treatment (*p* = 0.0054) (Figures 6H,I). And patients in the low-risk group became more susceptible to metformin (*p* = 0.0230) and nilotinib (*p* = 0.0550) (Figures 6J,K).

## Discussion

lncRNAs regulate different biological processes of cell metabolism and exhibit significant differential gene expression in liver metabolic diseases. HULC is an early confirmed lncRNA that is highly expressed in HCC [20]. It promotes the proliferation of HCC by upregulating the expression of peroxisome proliferator-activated receptor α and then activating the promoter of long-chain acyl-CoA synthase 1 (ACSL1). ACSL1 further promotes the production of acyl-CoA, thereby inducing abnormal lipid metabolism [21]. LncRNA-SOX2OT can enhance the metastatic performance of HCC and promote glucose metabolism [22]. FTx, MALAT1, and MOTAIR play significant roles in regulating cell metabolism [23–25]. Although some links between lncRNAs and cell metabolism have been revealed in HCC, a systematic analysis has not yet been conducted. In this study, 105 differentially expressed metabolism-related lncRNAs were identified in HCC. In addition, to facilitate and broaden clinical applications in different institutions, lncRNA pairs were constructed. A risk score model was built using the identified lncRNA pairs. The incidence and clinicopathological characteristics of HCC, such as pathological types and tissue types, are significantly different among different patients. The prognosis of patients in the same stage is also different. Therefore, there is a need for more awareness of the prognostic factors of HCC. The model divided patients into high- and low-risk groups, with statistically significant differences in survival between the two groups. The risk score had a higher prognostic capacity than that of age, sex, tumor stage, tumor grade, cancer status, and aneuploidy score.

Based on the literature search, we found that there were few reports on the underlying metabolism-related mechanism of most lncRNAs identified in this study, which are mainly determined as clinical prognostic factors for various tumors. Zhao et al. identified AC099850.4 as a top lncRNA of lncRNA-miRNA-mRNA competing triplets in ovarian cancer [26]. The LINC00239-based risk score model can predict the prognosis of HCC patients with cirrhosis [27]. Jiang et al. found MELTF-AS1 to be one of the most significant prognostic immune-related lncRNAs in clear cell renal cell carcinoma [28]. Wu et al. determined that AL606489.1, an autophagy-related lncRNA, could predict the outcome of lung adenocarcinoma [29]. Additionally, TMPO-AS1 regulates bladder cancer progression *via* the TMPO-AS1/miR-98-5p/EBF1 signaling axis [30].

Another innovative finding in this study was that patients in the high-risk group exhibited higher responses to gemcitabine and epothilone B, whereas those in the low-risk group may be more sensitive to metformin and nilotinib. The gemcitabine regimen enhanced the survival and disease-free survival rate of patients with HCC in clinical settings, and our findings may indicate patients who are most likely to benefit from this drug regimen. Epothilone is a 16-element macrolide, which is a secondary metabolite produced by myxobacteria [31,32]. The activity of epothilone B was three orders of magnitude higher than that of paclitaxel in cytotoxicity tests, and its multidrug resistance-inhibitory activity was approximately 100 times that of paclitaxel [32]. In HCC cell lines, epothilone B was found to be more potent than taxanes and doxorubicin, and thus, a clinical study examining its potential application in HCC is warranted [33]. Nilotinib is a BCR-ABL kinase inhibitor approved by the FDA in 2007 to treat patients with chronic or accelerated leukemia who are resistant to imatinib. The available evidence indicates that nilotinib can induce autophagy in HCC cells *in vitro* [34]. As shown in Figure 6B, patients in the low-risk group may has high oxidative phosphorylation level. It is reported that metformin could suppress tumor growth by inhibiting certain steps in the mitochondrial electron transport chain, which may explain the reason that patients in the low-risk were more sensitive to metformin [35]. Using the R package “pRRophetic” the clinical chemotherapeutic response to the above chemotherapy drugs was predicted, and the findings will be useful for clinicians to develop personalized therapies.

Although our findings might be statistically compelling, the conclusions were only derived based on the information obtained from the database. We could not experimentally verify the findings owing to a small number of cases of HCC with primary site resection in our institute. However, we plan to set up a specimen bank of HCC and have sought cooperation from other hospitals. Because this was an exploratory study, the application value of these findings needs to be further verified by multi-center and large-sample clinical studies, thereby clinically verifying our conclusions in the near future. Regarding an experimental design, the internal and external validity of the study determines its authenticity and universality of experimental conclusions. Regarding internal threats to the validity of this study, there might be a selection bias for patients with HCC in TCGA from the US population, which mainly included those with non-alcoholic fatty liver disease-related HCC. HBV-associated HCC is more common in East Asian countries. Regarding external threats to the validity of this study, different detection methods or detection platforms adopted by different institutions limit the application of the risk score model. Thus, we compared the values of two lncRNAs for lncRNA pairs and assigned them values according to their relative sizes, which might facilitate and broaden the clinical applications in different institutions.

## Conclusion

By screening differentially expressed metabolism-related lncRNA pairs in HCC, we constructed a 13-lncRNA pair-based risk score model. This model presented a better prediction of the outcomes than that with common clinicopathological characteristics, such as tumor stage, grade, and status and aneuploidy score. Moreover, the risk score model might help guide therapeutic regimens in the future.

## Data Availability

The datasets presented in this study can be found in online repositories. The names of the repository/repositories and accession number(s) can be found in the article/Supplementary Material.

## Supplementary Material

Supplementary Figure S1 Correlation of infiltrating immune cells and the risk score. **(A)** Correlation of infiltrating immune cells and the risk score of patients with HCC using various software. **(B–D)** The differentially infiltrating CD4^+^ Th2, T cell CD4^+^ memory, and macrophage M0 in the high- and low-risk groups.

Supplementary Table S1 2752 metabolic genes involved in the study.

Supplementary Table S2 Correlation of metabolic genes and lncRNAs.

Supplementary Table S3 105 differentially expressed metabolic related lncRNAs in HCC.

Supplementary Table S4 Value of lncRNA pairs of HCC patients.

Supplementary Table S5 The univariable cox regression analyasis of lncRNA pairs.

Supplementary Table S6 The risk score and risk stratification of HCC patients.

Supplementary Table S7 Correlation of risk score group and infiltrating immune cells.

Supplementary Table S8 List of drug sensitivity prediction of the high- and low-risk groups.

## Abbreviations

AUC, area under the ROC curve; GSEA, gene set enrichment analysis; HCC, hepatocellular carcinoma; HR, hazard ratio; LASSO, least absolute shrinkage and selection operator; lncRNAs, long non-coding RNAs; ROC, receiver operating characteristic; TCGA, the cancer genome atlas.
