# Supplementary figures and images for: Construction of a Metabolism-Related Long Non-Coding RNAs-Based Risk Score Model of Hepatocellular Carcinoma for Prognosis and Personalized Treatment Prediction
Source: Pathol Oncol Res. 2022 May 24;28:1610066. doi: 10.3389/pore.2022.1610066 (PMC9171512; doi:10.3389/pore.2022.1610066)

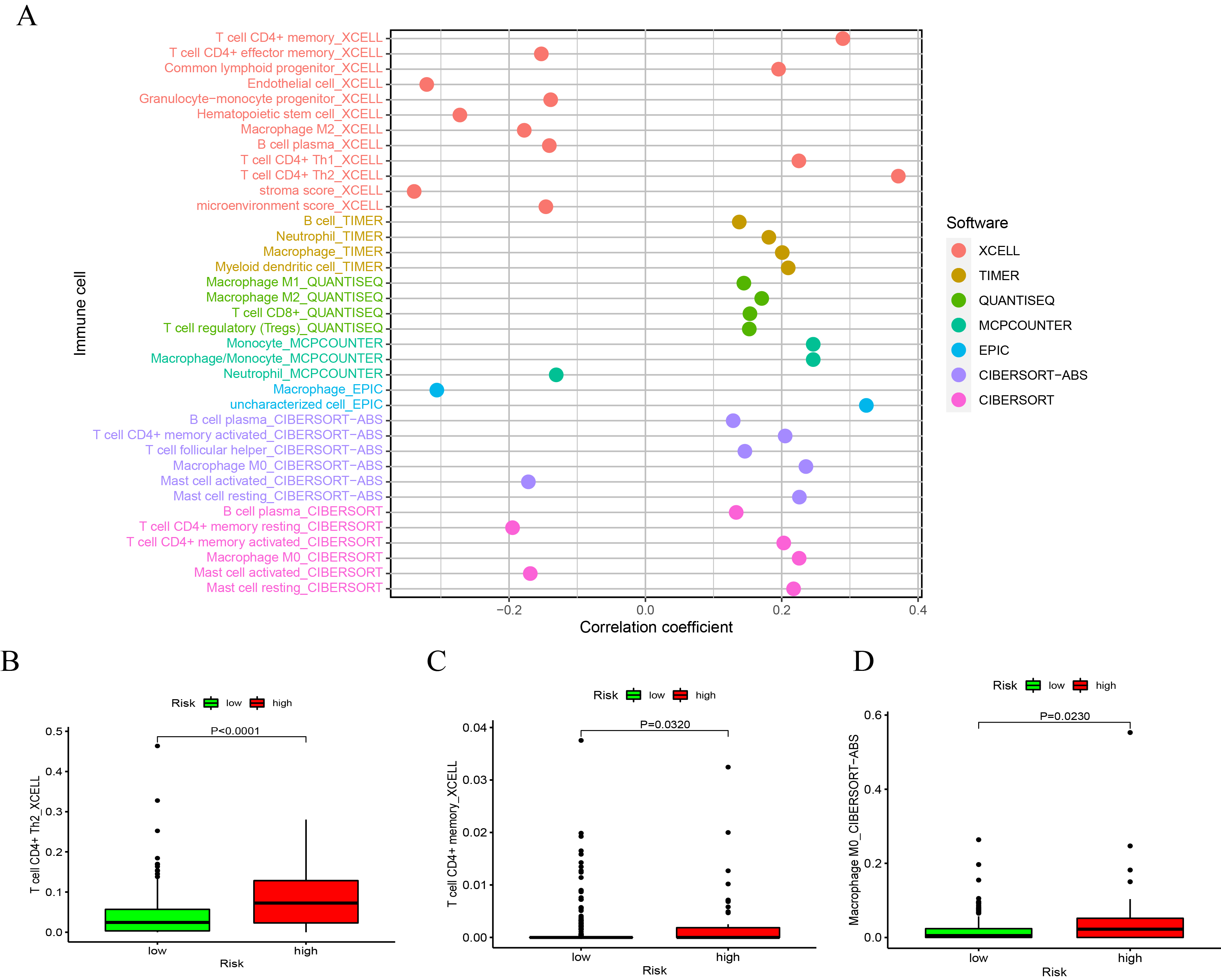

Supplement: Supplementary file 4 [file Image1.JPEG]
